# How Haptic Size Sensations Improve Distance Perception

**DOI:** 10.1371/journal.pcbi.1002080

**Published:** 2011-06-30

**Authors:** Peter W. Battaglia, Daniel Kersten, Paul R. Schrater

**Affiliations:** 1 BCS and CSAIL, MIT, Cambridge, Massachusetts, United States of America; 2 Psychology, University of Minnesota, Minneapolis, Minnesota, United States of America; 3 Psychology and Computer Science, University of Minnesota, Minneapolis, Minnesota, United States of America; Northwestern University, United States of America

## Abstract

Determining distances to objects is one of the most ubiquitous perceptual tasks in everyday life. Nevertheless, it is challenging because the information from a single image confounds object size and distance. Though our brains frequently judge distances accurately, the underlying computations employed by the brain are not well understood. Our work illuminates these computions by formulating a family of probabilistic models that encompass a variety of distinct hypotheses about distance and size perception. We compare these models' predictions to a set of human distance judgments in an interception experiment and use Bayesian analysis tools to quantitatively select the best hypothesis on the basis of its explanatory power and robustness over experimental data. The central question is: whether, and how, human distance perception incorporates size cues to improve accuracy. Our conclusions are: 1) humans incorporate haptic object size sensations for distance perception, 2) the incorporation of haptic sensations is suboptimal given their reliability, 3) humans use environmentally accurate size and distance priors, 4) distance judgments are produced by perceptual “posterior sampling”. In addition, we compared our model's estimated sensory and motor noise parameters with previously reported measurements in the perceptual literature and found good correspondence between them. Taken together, these results represent a major step forward in establishing the computational underpinnings of human distance perception and the role of size information.

## Author Summary

Perceiving the distance to an object can be difficult because a monocular visual image is influenced by the object's distance *and* size, so the object's image size alone cannot uniquely determine the distance. However, because object distance is so important in everyday life, our brains have developed various strategies to overcome this difficulty and enable accurate perceptual distance estimates. A key strategy the brain employs is to use touched size sensations, as well as background information regarding the object's size, to rule out incorrect size/distance combinations; our work studies the brain's computations that underpin this strategy. We modified a sophisticated model that prescribes how humans *should* estimate object distance to encompass a broad set of hypotheses about how humans *do* estimate distance in actuality. We then used data from a distance perception experiment to select which modified model best accounts for human performance. Our analysis reveals how people use touch sensations and how they bias their distance judgments to conform with true object statistics in the enviroment. Our results provide a comprehensive account of human distance perception and the role of size information, which significantly improves cognitive scientists' understanding of this fundamental, important, and ubiquitous behavior.

## Introduction

**Figure 1:**
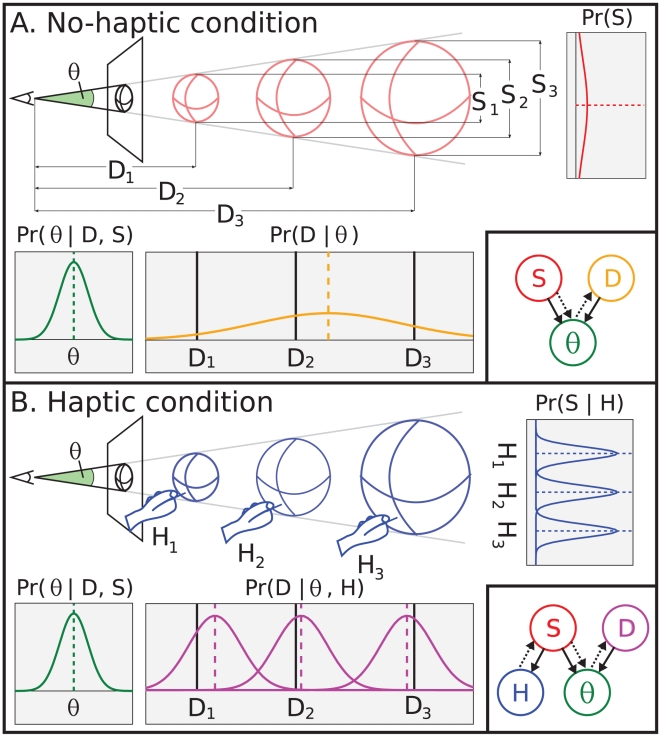
Task, model, and inference. **A. No-haptic condition.** The schematic shows a scene that contains a ball at some distance, and an observer who monocularly views a projected image of the ball (eye on left of image plane). In the absence of size information, the object's distance is ambiguous; e.g. the ball may be small and near (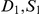), medium-sized and mid-range (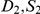), large and far (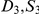), or anywhere in between, but still project to the same 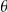. The lower-right inset is the no-haptic condition Bayes' net that shows the generative direction (black arrows), and information flow during inference (dotted arrows). 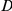 and 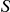 both influence 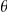 (black arrows), the likelihood of 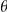 given 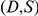 is the plot labeled “
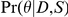
” on the left. Inferring the ball's distance means propagating prior information about 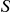 (“
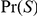
” plot on top-right) and 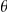 to form a posterior over 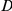 (labeled “
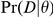
”). Notice that regardless of the true 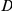 (i.e. 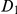, 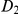, 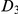, black vertical lines in posterior plot), the posterior over 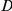 is the same, and is often positioned quite far from the true 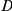. **B. Haptic condition.** The observer monocularly views an image of the scene and touches the ball beforehand to receive haptic size information, 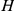. Though the image only constrains possible 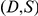 values to those consistent with 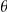, because 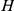 varies with 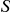 it constrains 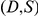 more and can disambiguate 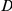. The lower-right inset is the haptic condition Bayes' net that shows the generative direction (black arrows), and the information flow (dotted arrows). The 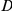 and 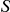 both influence 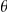 (black arrows), again the likelihood of 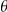 given 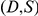 is the plot labeled “
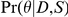
” on the left, but now the marginal posterior of 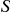 given 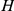 (plot labeled “
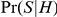
”) captures information about 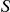. Inferring the ball's distance means propagating 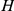 information, prior information about 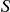, and 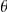 to form a posterior over 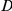 (labeled “
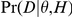
”). Notice that now different 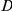 (i.e. 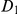, 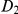, 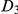, black vertical lines in 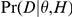) induce different posterior distributions (different curves in 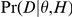), and each is positioned much nearer to the respective true 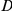.

The perception of distances by monocular vision is fundamentally ambiguous: an object that is small and near may create the same image as an object that is large and far (Figure 1A). More precisely, the monocular image size of the object (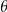, visual angle) does not uniquely specify the physical distance (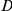), because 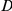 and the object's physical size (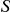, diameter) are confounded, 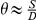. Subjectively we are not usually aware of this visual ambiguity because we perceive object distances unambiguously across a variety of conditions – this work examines how humans perform distance disambiguation by studying whether and how haptic size information is applied to these judgments. Despite previous evidence that adults [1] and infants [2] use object size information, like familiar size, to disambiguate (Figure 1B) the otherwise ambiguous visual information, debate exists [3], summarized by [2]. Recently, Battaglia et al. [4] reported that the brain merges image and haptic sensations in a principled fashion to unambiguously infer distance. Incorporating haptic size information is particularly interesting because it requires sophisticated causal knowledge of the relationship between distance, size, and the multisensory sensations available to the brain to overcome size/distance ambiguity.

Bayesian models provide the exact machinery needed to capture the size-distance perceptual ambiguity, the knowledge required to interpret noisy sensations, and how noisy sensations should be merged with prior knowledge to draw statistically sound perceptual estimates of object distances. This work uses Bayesian models to explicate, test, and confirm/deny a variety of hypotheses about the role of size information in human distance perception. Our results provide a significantly more comprehensive, quantitative account of the underlying computational processes responsible for incorporating size information into distance perception than any previous report.

We formulated a family of Bayesian perception/action models, whose model structure and parameters encoded different assumptions about observer's internal knowledge and computations. We analyzed Battaglia et al.'s [4] data within this context, and used statistical model-selection methods to infer the most probable model and associated parameters for explaining their data.

By committing to a full probabilistic model of observers' sensation, perception, and decision-making processes, we leveraged Battaglia et al.'s [4] data to uncover properties of: 1) the image and haptic sensory noise, 2) the observer's prior knowledge about size and distance, their causal relationship with the sensations, and how they are applied during perceptual processing, and 3) the decision-making strategy by which observers' perceptual inferences yielded psychophysical measurements. Important elements obscured from Battaglia et al.'s [4] original analyses were revealed: the present findings answer four key questions about how size influences human distance perception (described in Model section). Using a full observer model allows us to transcend simplistic debates about whether humans are “optimal vs. sub-optimal” by providing a more textured account of perceptual phenomena that quantifies the sensory quality, what internal knowledge is involved, how they are merged and exploited, and how decisions result. This allows vague questions like “Is perception Bayesian?” to be reformulated into more precise ones like “To what degree does the brain encode uncertainty and apply structured knowledge to perceptual inference?”

Our family of candidate observer models treat the world, observer, and observer's responses as one coherent interrelated physical system, which are represented in the models' structures and parameters using formal probabilistic notation. The fundamental assumptions are that world properties (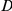 and 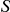) generate pieces of sensory evidence, or cues, (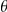, and the haptic size information 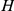), and the observer's perceptual process uses probabilistic (i.e. sensitive to various sources of noise and uncertainty) inference to compute the posterior distribution over the distance given sensory cues, 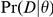 and 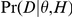 (Figure 1). The literature [5]–[8] reports many similarities between behavior prescribed by optimal Bayesian inference models, and humans' use of sensory cues, prior knowledge, and decision-making for perceptual inference. The perceptual task used by [4] is well-suited to Bayesian modeling because of important effects of uncertainty and especially the use of auxiliary information (in this case, 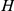) for disambiguating hidden causes (i.e. 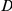). In fact, disambiguation of hidden causes using indirectly-related data is a key, beneficial feature of Bayesian inference, termed “explaining-away” [9]; we hypothesize that human distance perception in the presence of auxiliary size cues is consistent with probabilistic explaining-away.

Battaglia et al.'s [4] experimental task asked participants to intercept a moving ball, and treated their interception distances as perceptual distance judgments. Specifically, participants intercepted the ball as it moved at some distance, after a brief exposure to the ball that in some cases offered the ability touch the ball and feel its physical size and in other cases did not provide explicit size sensations. Our candidate observer models also make distance judgments using the sensory input available to human participants, so a direct comparison between human and model behaviors is possible.

We derived all our candidate models from a base, ideal observer model (IO) that contains internal knowledge about the distributions of sensory noise that corrupt the sensations 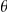 and 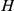, has knowledge about the prior distributions over 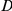 and 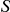, the relationship between 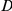, 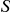, and 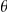, and the relationship between 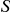 and 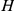 (Figure 1, lower-right insets, black arrows). In Bayesian parlance these pieces of knowledge fall under the rubric of *generative knowledge*, or background information about the data's generative process that can aid in inferring the underlying causes. The IO estimates 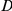 by computing 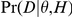 and selecting the 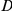 that maximizes it (“maximum *a posteriori*”, MAP, decision rule). This computation requires merging image-size and haptic cues, as well as prior distance and size knowledge, in a manner Bayes' rule prescribes to yield optimal information about 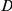 (Figure 1's caption illustrates the inference process). We formulated this IO, as well as the other candidate observer models by enumerating all combinations of the following hypothetical questions: 1) Does the observer use the haptic size cue?, 2) Does the observer know the haptic cue's reliability, and integrate the cue appropriately?, 3) Does the observer know the image-size cue's reliability, and integrate the cue appropriately?, 4) Does the observer perform MAP estimation, or rather estimate the distance by averaging a limited number of samples drawn from the posterior? The models were designed to allow standard model-selection methods to decide which hypothetical candidate model, and associated parameters, were best-supported by the experimental human data. Thus we were able to select the most accurate hypothesis, among the field we pre-specified, as the best explanation for how human distance processing uses size information. Moreover, we compared the resultant parameter estimates with measurements reported by other studies, and found they conform with previous findings regarding perception's computational dynamics, which provides independent verification of our conclusions' validity.

Our results indicate humans incorporate haptic size information for distance perception, consistent with Bayesian explaining-away. We also found that all but one participant underestimated the haptic cue's reliability (specifically, they overestimated its sensory noise variance) and integrated the haptic information to a lesser degree than the IO prescribed, similar to the human underuse of auditory information for spatial localization reported by [10]. We found that participants' priors over size and distance were comparable to the experiment's actual random size and distance parameter distributions, implying participants applied knowledge of probable stimulus parameters in their perceptual processing (possibly learned or assumed during the experiment). Last, the sample-averaging estimation model, as opposed to the MAP-estimator, best-accounted for participants' distance judgments, a finding consistent with a growing body of results from perceptual studies that suggest perceptual judgments result from posterior sampling processes [11]–[13].

## Model

The observer models have three components: 1) the *sensation model* describes how the distal stimulus determines the proximal stimulus, 2) the *perception model* describes how the distal stimulus is inferred from the proximal stimulus, 3) the *decision-making model* describes how the inferred distal representation guides action.

### Sensation model

The scene properties relevant for object distance perception are the object's physical distance and physical size; the relevant sensory cues they generate are visual angle and felt (“haptic”) size. As noted in the Introduction, visual angle is proportional to the ratio of size and distance; so, taking the 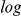 of each of these variables transforms this relationship into a linear sum (below). Our sensation model uses this 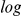-transformed representation for two reasons: 1) Weber-Fechner phenomena support a noise model in which the standard deviation linearly scales with signal magnitude (which can be accomplished with independent noise in log-coordinates), and 2) this 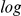-linear approximation is analytically tractable, as we will show. So we assume a linear Gaussian model, meaning the scene properties are *a priori* Gaussian distributed, and the sensory and motor noise are additive, zero-mean Gaussian, and the sensory generative process is linear, in the log domain.

Log-distance, log-size, log-visual angle, and log-haptic size are represented as: 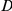, 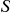, 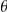, 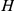, respectively. The relationship between 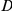, 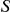, and 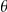 (by “small angle approximation” to 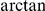) is:
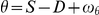
and between 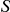 and 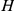 is:
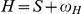
where 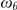 and 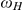 represent image-size and haptic sensory noise with standard deviations (SDs) 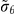 and 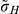, respectively. The 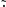 notation indicates that the parameter represents a *property* of the scene; this is distinct from the observer's *knowledge* about the scene, defined in the next section with no tilde.

It follows that the distribution of sensory cues conditioned on the scene properties are:
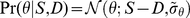
(1)

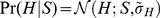
(2) We assume observers' internal prior probabilities over 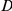 and 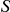 are:
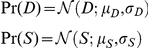



### Perceptual model

Battaglia [14] derives model observers for perceptual inference in linear Gaussian contexts under a variety of assumptions – we co-opt the “explaining-away” derivations (Sec. 3.4 in [14]) for the current size/distance perception context. All model observers are assumed to use their knowledge of the world, i.e. the sensory noise (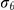 and 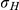) and prior distributions (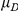, 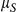, 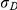, and 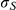), to compute beliefs about 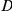. These beliefs are represented as the posterior distribution, 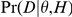 (which is Gaussian):
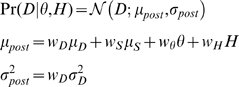
(3)where,
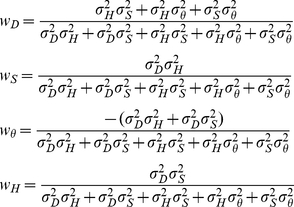
(4)


For those familiar with “standard” cue combination, Eqs. 3 and 4 are similar to the “optimal cue combination” formulae in [15], and in fact by looking closely at the Bayes' net in the lower right of Fig. 1B, one can see that the subgraph composed of variables 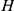, 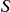, and 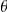 represents the standard two-cue “cue combination” situation. However, our present situation is distinct from [15] because we focus on data fusion in conditions where one cue (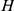) is only indirectly related to the desired property (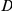) by its ability to disambiguate another cue (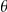). The intuition for the weights in Eq. 4 is as follows. Because 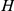 provides information about 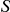 to improve inference of 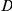, the numerator of 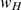 assigns sensory cue 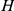 more influence when prior knowledge of 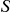 and 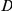 are weaker (higher 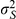 and 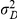). Similarly, 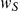
's numerator dictates that 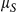 is more influential when information about 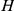 is weaker (higher 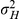). Interpreting 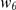 is less straightfoward, but essentially holds that when information about 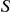 is poor, because both the prior over 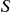 and sensory cue 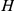 are weak (higher 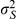 and 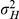), then 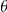 is more exclusively influential for inferring 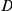, whereas if either prior knowledge about 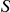 or sensory cue 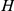 are strong, 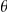 and that 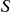 information jointly guide inference of 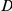. Last, 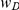
's numerator assigns stronger influence to prior knowledge of 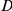 only when the sensory cues and prior knowledge of 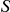 are weak.

Human observers who *do* use 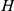 for distance perception are modeled above by Eq. 4. The hypothesis that observers *do not* use 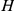, either because 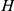 is unavailable or because they are not capable, is formulated:
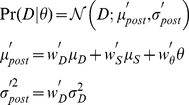
(5)where,
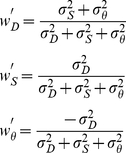
(6)


Eq. 5 is algebraically equivalent to taking 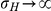 in the formulation in Eq. 4. Whether humans do (Eq. 3) or do not (Eq. 5) use 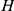 to make distance judgments is the first of our hypothesis questions (see Table 1). Also, whether humans know the true sensory noise magnitude i.e. whether they use 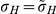 vs. 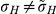, and/or 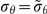 vs. 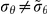, are the second and third of our hypothesis questions (Table 1).

**Table 1:** Candidate model list.

Model #	Q I	Q II	Q III	Q IV
1	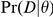	n/a	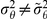	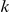
2	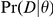	n/a	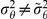	MAP
3	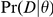	n/a	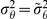	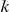
4	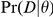	n/a	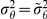	MAP
5	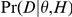	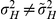	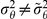	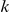
6	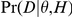	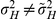	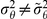	MAP
7	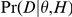	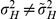	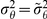	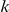
8	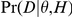	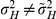	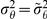	MAP
9	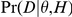	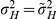	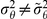	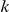
10	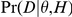	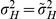	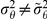	MAP
11	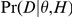	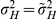	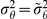	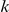
12	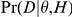	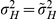	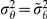	MAP

The candidate models encode possible answers to the four questions as follows. Q I: “
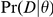
”, haptic information is never integrated vs. “
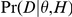
”, haptic information is integrated when available. Q II (which is only applicable to the 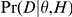 answer for Q I): “
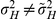
”, internal knowledge of haptic reliability is incorrect vs. “
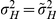
”, internal knowledge of haptic reliability is correct. Q III: “
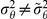
”, internal knowledge of image-size reliability is incorrect vs. “
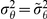
”, internal knowledge of image-size reliability is correct. Q IV: “
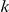
”, posterior samples are averaged to form distance judgments vs. “MAP”, MAP estimates are used to form distance judgments.

### Decision-making model

The model observer uses beliefs about 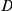 to select a position at which to intercept the moving ball. We assume that participants attempt to minimize the difference between their judged distance and the true distance, which for Gaussian distributions may equivalently correspond to minimizing a MAP, mean-squared, or symmetric Heaviside loss functions. However accessing their perceptually-inferred information about 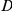 is not necessarily trivial: we consider that they may select the maximum probability 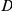, i.e. 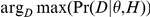 (or 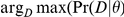)), as their judgment of distance, or instead draw a number, 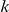, of independent samples from 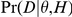 or 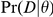 and compute their sample mean as a 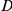 judgment, is the fourth (and last) of our hypothesis questions (Table 1). These distinct models may imply different neural representations for posterior beliefs about distance, which we address in the Discussion.

Additionally our models all include an element of motor noise, the small degree of error between judged 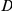 and the experimentally-measured 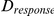, due to motor imprecision when performing an interception. For consistency with known parameters of motor control, we selected an additive, Gaussian motor noise term 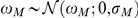, that was added to the distance judgment to form 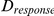.

### Full observer models

We combine the sensation, perception, and decision-making models described above to define a set of coherent model observers that input sensations, combine them with internal knowledge to form beliefs about distance, and form decisions that are output as interception responses in the experimental task.

By varying the models' structure and parameters we encoded the four hypothesis questions in the Introduction (subsequently referred to as “Q I, II, II, IV”) to form the candidate observer models (Table 1):

- I) Does the brain integrate haptic size information for distance perception (i.e. do they use 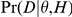 when possible, or 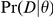 exclusively)?
- II) Does the brain have accurate knowledge of the haptic cue's noise magnitude and incorporate 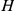 in proportion to its reliability? (i.e. do they use 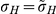 or 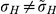)?
- III) Does the brain have accurate knowledge of the visual image-size cue's noise magnitude and incorporate 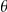 in proportion to its reliability (i.e. do they use 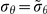 or 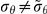)?
- IV) Does the brain select MAP distance estimates, or average 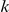 samples from 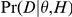 or 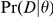
?

In total 12 distinct candidate models spanned the possible combinations of the four questions (the reason the total is 12, instead of 16, is because for candidate models that do not include the use of haptic information [Q I], the question of whether the observer knows the haptic cue noise magnitude or not [Q II] is inconsequential and those models are redundant).

### Human data methods

#### Ethics statement

All participants gave informed consent in accordance with the University of Minnesota's IRB standards.

#### Stimuli and task

**Figure 2:**
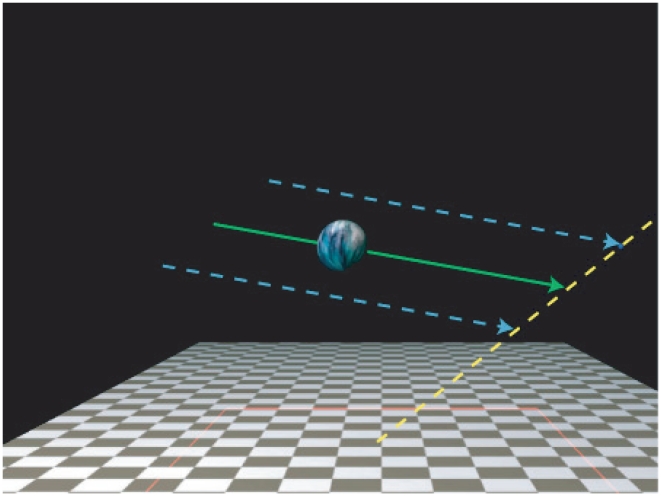
Experimental stimulus screenshot. The overlaid lines were not visible to the experimental participant, but depict various task elements; they are not drawn from the participant's viewpoint, but rather from a viewpoint elevated above the observer's head so they can be distinguished from each other the participant's viewpoint intersected the constraint line). They are: the constraint line (yellow dotted line), the ball's true movement path (green solid arrow), and ambiguous movement paths in the no-haptic condition (blue dotted arrows). The point at which the green arrow intersects the constraint line is the crossing distance. The points at which the blue arrows intersect the constraint line represent distance misjudgments. The participant's hand position was indicated by a 3 mm diameter blue sphere.

Participants sat in a virtual reality workbench capable of presenting monocular visual (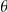) and haptic (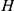) stimuli (Figure 2 shows a stimulus screenshot). They held a small, stylus probe connected to a robot arm that presented forces and recorded their hand movements; the hand/stylus position was graphically depicted in the visual scene as a small 3 mm *stylus sphere* (see [4] for full experimental details). They performed 1280 trials across 4 days, where each day was composed of 4 blocks of 80 trials; the first day of trials was treated as training, and excluded from further analysis, resulting in 960 total experimental trials. There were two types of trials, *no-haptic* and *haptic*, randomly interleaved in equal proportions (480+480 = 960), which determined the type of exploration phase (described next).

Each trial was divided into two phases: *exploration* and *interception*. During the exploration phase, a ball with random diameter between 14 and 42 mm appeared at a random position in the virtual scene between 300 and 640 mm distance, at an angle between −8.5 and 8.5 degrees visual angle on the horizontal plane that intersected the eyes, and remained still. On no-haptic trials, participants viewed the ball but were not able to touch it, on haptic trials they touched the ball with the stylus and received haptic force feedback consistent with the ball's physical diameter [4]. Once they were satisfied with the exploration they depressed a mouse button to complete the exploration phase and begin the interception phase; the exploration phase was forced to last a minimum of 1 second, and additionally in haptic trials participants had to touch the ball to end the exploration phase.

Both trial types' interception phases proceeded identically. First the robot arm moved the hand to a position on the right side of the scene, and began to impose a continuous constraining force that limited the hand's position to a fixed ray that began at their eye and extended toward the ball's (future) movement path. Simultaneously the ball was repositioned at a random position on the left of the scene at a distance between 1000 and 1500 mm and an angle between −17 and −5 degrees on the horizontal plane. This rendered any distance information gained during the exploration phase irrelevant (and thus non-useful for subsequent distance judgments), but the ball's size was kept the same. Once ready, the participant again depressed the mouse button and the ball began to move toward the constraining line with a random speed between 250 and 375 mm/s. The ball's trajectory crossed the constraining line at a random point between 300 and 640 mm from the participant's eye (termed the *crossing distance*) and continued out of the scene; the total travel time was between 1.3 and 4.8 seconds. Participants were instructed to place the stylus' tip at the crossing distance, and we recorded this position at the time the ball crossed the constraining line as the *judged distance*, which was used for the subsequent data analysis as an indication of the participant's perceived distance. Participants received haptic feedback regarding their accuracy: if the judged distance was within 32 mm of the crossing distance the stylus received an impulse consistent with a momentus collision and the finger visual stylus sphere pulsed green momentarily, otherwise no collision was felt and the stylus sphere pulsed red. At this point the trial ended and a new trial began immediately.

#### Participants

6 university students, ages 21 to 30, participated in the study. All had normal or corrected-to-normal vision, and normal motor abilities. 5 participants were naive to the purpose of the study, 1 was an author; the author's data was statistically indistinguishable from the others'. All participants gave informed consent in accordance with the University of Minnesota's IRB standards.

### Analysis

First, we describe how the model observers predict responses in the experimental interception task and illustrate responses produced by each model. Second, we describe how the model's parameters were inferred given each participants' response data. Third, we show how we computed the human data likelihood under each model and how we quantitatively compare them to determine which model provides the best account of the human data.

#### Generating model predictions

**Figure 3:**
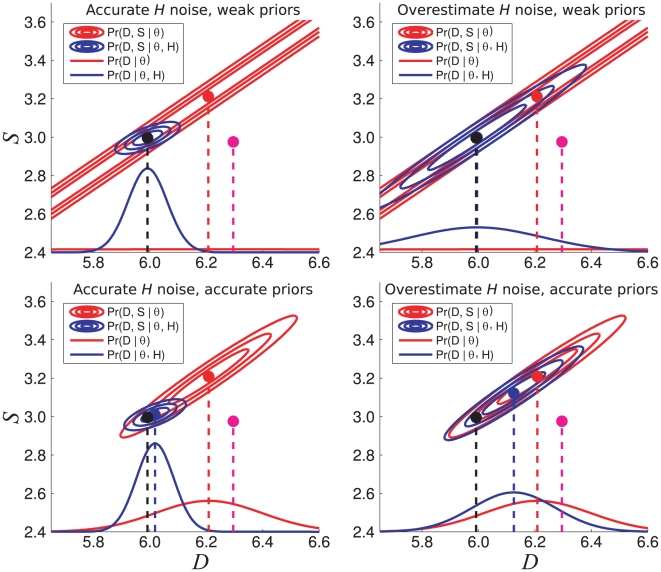
Perceptual model. Posterior distributions over 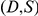 given sensory input 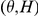 are depicted. The true values of 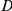 and 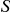 are 6.0 and 3.0, respectively. The red curves are when the haptic information is not incorporated, the blue curves are when it is incorporated. The curves on the bottom are the joint distributions marginalized over 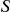, to yield marginal posteriors over 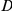. The dotted vertical lines are the posterior means. The black dot is the true 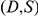 values, the purple dot is the 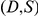 prior mean. The top row is an observer who uses weak priors (high 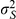 and 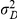) and the bottom row is an observer who uses accurate priors (lower 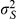 and 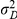). The left column is an observer with accurate knowledge of the haptic noise (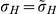) and the right column is an observer with inaccurate knowledge (overestimated) of haptic noise (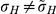). Notice that by using haptic information, the mean of the posterior becomes more accurate and the variance decreases. When prior information used, bias is introduced in that the means become less accurate, however the posterior variance decreases. Also notice that when the haptic cue noise is inaccurate the observer's posterior shifts toward the no-haptic integration observer.

We can simulate the sensory model by fixing 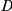 and 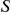 and sampling predicted 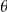 and 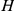 values. Beliefs about 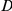 are represented by Gaussian posterior distributions in our model (Eqs. 4 and 5), whose means and variances depend on the assumptions encoded by Q I-III. Figure 3 shows posteriors over 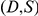 (contours) and 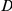 (density functions on bottom) given 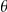 when haptic information is not integrated (red) and 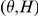, when haptic information is integrated (blue). When prior information is weak (top row), the sensory cues dominate, and the posterior variance is high; when no haptic information is incorporated the posterior is ambiguous, many 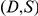 values are consistent with 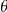. When prior information is strong (bottom row), prior bias is introduced (ie. black and blue posterior mean lines separate) but on average 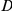 judgments are more accurate because sensory noise is mitigated by prior knowledge, and the posterior distribution's variance shrinks. Weak priors are approximated by Gaussians with very high variance.

**Figure 4:**
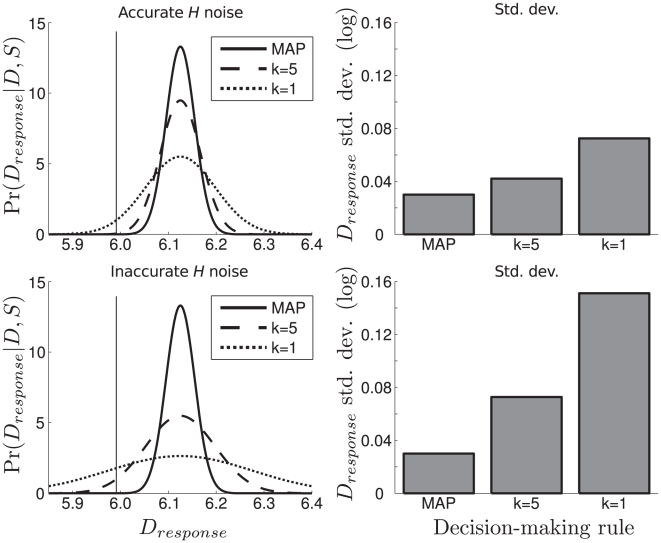
Decision-making model. The left column shows model observers' response distributions given input 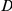 and 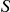 values 6.0 and 3.0, respectively. The top row is when accurate haptic noise knowledge is used, the bottom row is when inaccurate haptic noise is used. The solid vertical lines are the true values of 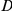. The solid distribution is an observer that uses the MAP estimate to make a distance judgment, the dashed distribution is an observer that draws 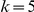 posterior 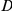 samples and averages their values, the dotted distribution is an observer that draws 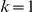 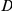 sample. The right column shows the corresponding SDs of the distributions in the left column. Notice that the sampling observers have less precise response distributions than the MAP-responder, and averaging over fewer samples yields less precise responses.

Each model observer's responses can be predicted given input 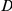 and 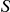 values. Q IV distinguishes between decision-making strategies. Figure 4 depicts an empirical distribution of model observers' responses, under MAP estimation as well as sample-averaging estimation (with 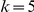 and 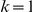).

#### Posterior over model parameters

To test each model's account of the human data, our analysis inferred each model's parameters, 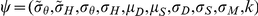, given the participants' experimental responses, 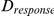; the posterior distribution is 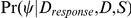. (Note, 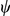 varies depending on the model, for instance the MAP decision-maker does not have a 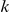 term, the observer that does not use haptic information does not have 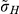 or 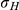 terms, etc.) The likelihood functions were straightforward to compute: because we defined our model observers' entire sensation-perception-decision sequence as a probabilistic generative process, we could compute the participants' response likelihoods given the input stimuli, 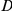 and 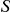, for each model.

We constructed the model observers' response likelihood functions by first considering the observers' inferences about 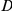, which are summarized by the model's posterior parameters, 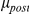 and 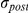. So, 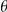 and 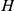 were treated as random variables with distributions in Eqs. 1 and 2, and the likelihood of posterior means given 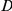 and 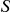 when haptic size information is used is
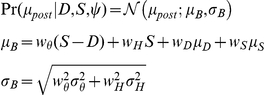
with 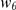, 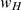, 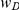, and 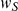 from Eq. 4. The likelihood of posterior means when haptic information is not used is
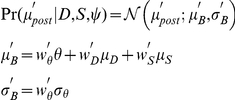
where 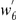, 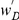, and 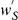 are from Eq. 6.

Model observers' 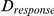 were based on their posterior distributions: for MAP, only 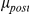 is needed, but for sample-averaging, 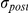 is also involved. And, motor noise 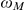 was always added.

A MAP observer's 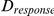 likelihood is,
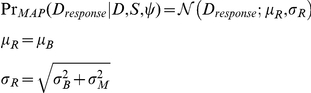
And a sample-averaging observer's likelihood is,
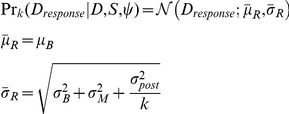
where 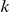 is the number of samples that were averaged.

The prior over 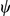, 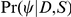, was chosen to be uniformative, we assumed uniform prior distributions over a very large range of possible parameter values.

#### Comparing models with humans

For each model, for each participant, we wished to find the most probable parameters given the data we measured. However, because the models have numerous parameters and are nonlinear, optimizing for the parameters is difficult. Also, knowing the *posterior distribution* of parameter values is preferred to optimizing the parameters, because optimizing is subject to overfitting, while the posterior distribution implicitly captures the quality of the fit. We used Markov Chain Monte Carlo (MCMC) [16] to approximate samples from the posterior, then within each participant compared how well each model explains the data by computing a standard model “goodness” metric called DIC [16]. DIC rewards predictive power and penalizes model complexity; lower DIC scores mean better fits. DIC is similar to related model goodness metrics, like Akaike Information Criterion and Bayesian Information Criterion, but is especially suited to MCMC output. So, for each participant the model with the lowest DIC score provided the best account of the data, in terms of explanatory power *and* parsimony.

Battaglia et al.'s [4] interception experiment collected each participants' 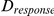 measurements given 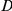 and 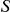 in two conditions, no-haptic and haptic. In the no-haptic trials, all models used Eq. 5 to draw perceptual inferences. In the haptic trials, models that integrate haptic information (Q I) used Eq. 3 while models that did not integrate haptic information again used Eq. 5. Different trials were treated as independent, and we computed the total experimental likelihood, 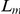, for each model 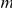 as the product of each trial's likelihood, 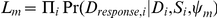, where 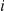 is the trial number.

The likelihood (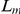) and prior (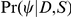) terms allowed us to draw MCMC-simulated 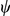 parameter samples from the posterior 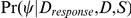 (Metropolis-Hastings specifically). For each model, 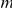, for each participant, we drew a set of 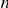 simulated MCMC parameter samples, 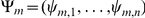; we ran 360 parallel chains with 15,000 “burn-in”, throwaway samples followed by 6,000 stored, valid samples (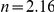 million valid samples).

DIC scores are based on the *deviance*, 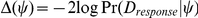, of the MCMC posterior model parameter samples, 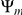. DIC sums two terms, the expected deviance 
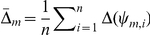
, and the model complexity 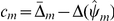, resulting in 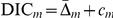. Here 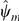 is a “good” parameter estimate for the model, usually computed as the mean, median, or other central tendency statistic, of the set 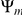; we used a robust mean to compute 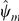, which eliminated outlier parameter values. Significance metrics with respect to DIC scores, in the traditional frequentist sense, has not been exhaustively studied, however DIC is a Bayesian analog to Akaike Information Criterion (AIC), and [17] suggest that models with AIC/DIC greater than 3–7 above the “best” model are “considerably less support[ed]”. We chose to report DIC differences greater than 10 as significant, and 15 as “highly significant”. We computed each model's DIC score separately for each participant, to quantitatively select the best explanation of the participant's pattern of responses.

## Results

### Human performance

**Figure 5:**
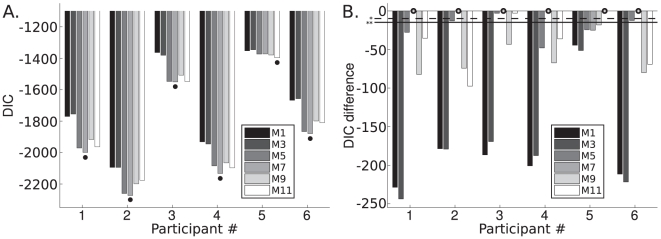
Models' DICs. **A.** DIC scores for each sample-averaging model for each participant (lower is better); significance (*) is defined as 10 DIC difference, high significance (**) is 15 DIC difference. Each cluster of 6 bars are models' 1, 3, 5, 7, 9, 11 DIC; the 6 clusters of bars are the 6 participants. **B.** Differences in DIC between the best model (indicated by circle on x-axis) and the other models for that participant (higher values mean closer DIC score to the best model). The reason only the odd models, which correspond to the sample-averaging decision-making procedures, are shown is because their DICs are substantially better than their MAP counterparts.

The central result of our study is quantitative selection of the model that best explains the data, which we determine by comparing the models' DIC scores, to answer the four hypothetical questions posed above. Figure 5A shows raw DIC scores, and Figure 5B shows the difference between the best model's DIC (indicated by circle on x-axis) and the other models' DIC scores. We defined DIC significance as described in the previous subsection: models whose DIC differed by greater than 10 were deemed “sigificantly” different (dashed horizontal line and * in Figure 5B) and greater than 15 deemed “highly significantly different” (solid horizontal line and ** in Figure 5B); this is a conservative modification of the criteria mentioned in [17].

We found that all participants incorporate haptic size information to make their distance judgments (Q I). Also, we found 5 of 6 participants misestimated their haptic size noise and thus incorporated the haptic information less than optimally prescribed, while one participant applied the haptic cue in proportion to its reliability (Q II); the following section addresses the nature of the misestimation. All participants incorporated the visual image-size cue optimally, in accordance with its noise magnitude (Q III). All participants used a sample-averaging strategy over MAP decision-making (Q IV). With respect to Q IV, the DIC scores were always worse for the MAP model versus the sample-averaging model, by an average DIC difference of 129 (Figure 5), so we exclusively focus on the sample-averaging models (odd numbers) for the remaining discussion. Figure 5 depicts each participant's DIC scores for each sample-averaging model, the left graph shows the absolute DIC values and the right graph shows the differences between best model DICs and the other models' DICs. Participant 6 was an author. Participant 3's DIC differences between Model 7 and Models 5 and 11 was not significant under our conservative criteria, however Model 7 was still better by DICs of 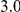 and 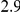, respectively, which is considered marginally significant under typical uses of AIC/DIC [17].

Participant 5, the only participant whose DIC favored the hypothesis that the haptic noise magnitude was correctly known (Q II), had the worst DIC scores across participants, as well as substantially different parameter value estimates from the other participants (see next paragraphs). Upon closer inspection of participant 5's data, it was qualitatively the noisiest: in Battaglia et al.'s [4] simple regression analysis of this data their statistical analysis determined participant 5's data was so significantly different from the other participants' that it ought to be excluded as an outlier. The reason we included it in the current analysis was to determine whether there was still some patterns the previous analysis had not detected. Though the parameters still yield meaningful values, because of the major differences between raw DIC scores, the DIC-favored model, the parameter value estimates, and the general noisiness of the response data, we strongly suspect this participant either was not focusing on performing this task, was randomly selecting answers on a large fraction of the trials, and in general should be distinguished in further analysis due to these aberrations: so, we report participant 5's parameter estimates separately from the other “inlier” participants.

**Figure 6:**
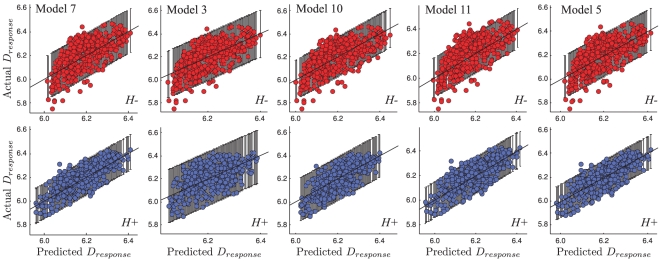
Effect of model on predicted vs. actual 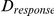. Each plot shows the predicted 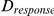 (x-axis) versus the actual 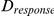 (y-axis) across several different models for Participant 1. The black diagonal line represents perfect correspondence between mean predicted and actual 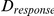 values; the black error bars represent the 95% confidence interval of the model's predicted 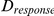. The colored dots represent all the actual measured 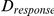 values; the top row represents no-haptic trials (red, labeled 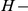), the bottom row represents haptic trials (blue, labeled 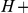). Each column is a different model (numbered along top row, see Table 1), all predicted 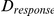 are based on the inferred, MCMC-expected parameters for that model. Model 7 (*column 1*) is the best model for the inlier participants, and the others are variations of Model 7 with one difference: Model 3 (*column 2*) does not use haptic cues; Model 10 (*column 3*) uses MAP estimation instead of sampling; Model 11 (*column 4*) describes an observer that knows the haptic noise accurately; Model 5 (*column 5*) describes an observer that uses inaccurate knowledge of the image noise. When comparing Model 7 to the worse-fit models, consider the correspondences between predicted and actual 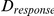 means and variances, ie. how neatly the actual data falls within the predicted bounds. Also, for Model 3 note that the 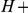 predictions are not as constrained as Model 7. And, notice Model 10 cannot jointly predict the higher variance in the 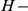 actual 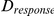 and the lower variance in the 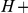 actual 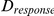. Models 11 and 5 have predictive accuracy nearer that of Model 7, but still have worse fits as summarized by the DIC scores (Figure 5). (Slight differences between Models 7 and 3's 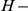 predictions are due to stochasticity in the MCMC sampling procedure.)

Figure 6 shows Participant 1's model-predicted 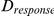 compared against the actual 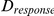 values, for the best model, 7, as well as several that differ by one assumption (Table 1). The spread in the dots is due to sensory noise and the random posterior sampling process, how neatly the actual data falls within the ranges predicted by a particular model (black error bars) is indicative of the model's explanatory quality. Notice the pattern of more varied no-haptic vs. haptic 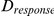 in Models 7, 11, 5, a direct prediction of the sampling models over MAP. Though MAP decisions incur more bias in the no-haptic condition, they actually have less trial-to-trial variance than in the haptic condition. This is due to the fact that the prior does not vary between trials, while the more informative haptic cue does.

A possible concern is that participants learned to use the haptic cue during the course of the experiment, and that the weak DIC scores of Models 1–4 in comparison to Models 5–12 actually reflect the effects of associative learning rather than knowledge the participants brought into the experiment. We evaluated this possibility by performing the same DIC analysis on data from only the first day to test whether Models 5–12 were still favored over their 1–4 counterparts. The results unequivocally confirm the results on the data from the final 3 days above: for every participant, the DIC analysis across the models shows that the no-haptic models (1–4) have worse DIC scores than their haptic model counterparts (5–12). The best no-haptic models' DICs are below the best haptic models' DICs by margins of {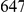, 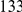, 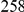, 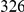, 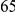, 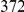} for Participants 1 through 6, respectively. In fact, removing the sampling models, even the no-haptic models (1 and 3) with the best DIC scores still have worse scores than the haptic models (5, 7, 9, and 11) with the worst DIC scores. This firmly supports conclusion that the haptic cue is used even on the first day of trials.

Though it might seem that given the 6 to 10 “free” parameters in our general observer model, we could “fit” any data, we are actually inferring the best parameters and using the posterior's expected values rather than the most probable a posteriori parameters. Moreover DIC acknowledges the possibility for overfitting and counters it by penalizing overfits through the complexity term, thus affirming that the chosen model's structure and parameters are accurate and robust explanations of the humans' judgments. Moreover because we encoded different hypotheses within the models we could clearly distinguish those hypotheses best-supported by the data. Lastly, despite the possibility that we *could* fit a variety of data, the remainder of this section shows that the individual inferred parameter values are consistent with known perceptual parameters measured in other studies.

### Inferred model parameters

A secondary result of this work, beyond providing answers to the 4 hypothetical questions, is that the inferred parameter values (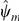) our analysis yielded can be meaningfully interpreted. Though there is no guarantee that the inferred parameters are unique, they offer an indication of what the analysis finds probable. All reported parameters are MCMC expections, from which we compute means
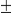
SEs across participants and report the values in log coordinates. First, we present the SDs in terms of Weber fractions for the sensory noise, with discrimination thresholds corresponding to 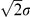.

The image-size noise SD, 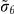, and assumed noise SD, 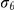, were coupled in the best-fit models (7 and 11) for all participants. Their values correspond to Weber fractions of 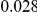 to 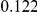 (mean
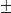
SE of 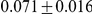) for the inlier participants, and 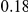 for participant 5. This is comparable to the Weber fractions of 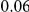 measured in humans by [18] for parallel line separation discrimination, and 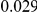 by [19] for line length discrimination. Because our task did not involve interval-wise discrimination of pairs of stimuli, but rather absolute perception, it is to be expected that our noise magnitudes will be slightly higher.

The haptic noise SDs, 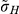, and assumed haptic noise SD, 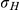, were uncoupled in the inlier participants' best-fit model (7), and coupled for participant 5's best fit model. The inlier participants' haptic noise SDs correspond to Weber fractions of between 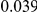 and 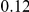 (mean
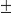
SE of 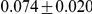), and 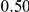 for participant 5. A Weber fraction of 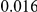 was measured in humans by [20] for haptic size discrimination of objects between 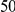 and 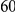 mm in width using a similar haptic stimulus presentation apparatus, but with two fingers gripping the object rather than one finger probing the size. Because two fingers are likely to provide a more precise size measurement and because their participants performed interval discriminations of pairs of objects, our somewhat elevated Weber fraction are reasonable values. The inlier participants overestimated their haptic noise SDs, with their assumptions corresponding to Weber fractions of 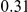 to 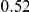 (mean
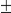
SE of 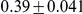). The consequences of overestimating haptic noise are that the observers do not achieve the level of disambiguation possible by fully incorporating the haptic cue, and apply prior knowledge about the ball's size and distance relatively more heavily (Figure 3).

Our analysis provided information about the observer models' prior knowledge, and found it strikingly similar to the sample statistics of the experimental stimuli's distances and sizes, with slightly higher SDs (remember the stimuli were uniformly distributed in the mm domain). The mean
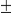
SE estimated prior distance mean and SD parameters, 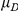 and 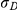, across all participants were 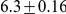 and 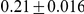 log-mm, respectively; the experimental distance mean and SD were 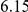 and 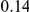 log-mm, respectively. The mean estimated prior size mean and SD parameters, 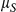 and 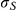, across participants were 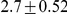 and 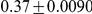 log-mm, respectively; the experimental size mean and SD were 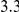 and 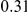 log-mm, respectively. This indicates participants learned the range of possible stimuli presented in the experiment and applied that knowledge toward improving their judgments, to the effect of lowering the posterior variance (Figure 3). To further investigate the source of participants' prior knowledge, we ran our full analysis on only the first day of participants' trials, to measure what difference between inferred parameters exist between early and later in the experiment. We found that participants' first-day priors for 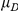 and 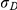 were 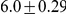 and 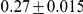 log-mm, respectively; and, participants' first-day priors for 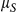 and 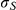 were 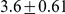 and 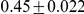 log-mm, respectively. So, the prior means did not shift significantly (in terms of SE interval overlap), but the prior SD values did. It appears that participants rapidly learned the prior means, which are more easily estimable from experience and also may be assumed to some extent (the true prior distance mean is at the center of the virtual workbench, and the balls' sizes were directly observable in haptic condition trials). However, participants appeared to use more diffuse prior 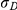 and 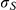 parameters early in the experiment, which is consistent with making weaker prior assumptions about the range of distance/size variation (top row of Figure 3).

Our analysis provided estimates of participants' motor noise SD, whose mean
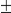
SE across participants was 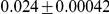 log-mm, which amounts to a SD of 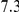 mm at a reach distance of 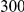 mm, and 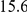 mm at reach distance of 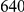 mm, the extremal distances presented in the experiment. A value of 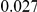 log-mm was reported [21] under similar reaching conditions.

The sample-averaging models generally outperformed the MAP estimate models (Q IV) with respect to DIC scores. The inlier participants had 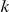 values between 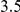 and 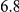 (mean
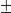
SE of 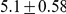), and participant 5's 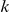 estimate was 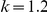. Of course in our model 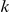 must be integer-valued, but these real valued estimates are robust means across our MCMC analysis samples. An alternative interpretation of 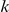 is that it is an exponent applied to the posterior distribution, from which one sample is then drawn after renormalizing. For a Gaussian distribution, because 
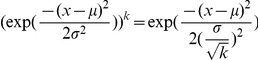
, drawing 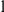 sample, 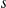, from 
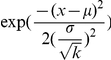
 yields a Gaussian-distributed: 
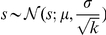
. And, drawing 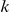 samples, 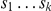, from the unexponentiated 
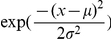
 and averaging yields a sample mean with the same distribution: 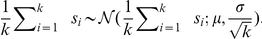



Between the DIC analysis and the validity of the inferred parameters, we conclude that model 7 is both structurally and parametrically accurate. This strongly supports model 7 and its encoded hypotheses as a coherent computational account of the underlying processes responsible for size-aided distance perception.

## Discussion

We conclude that humans can use haptic size cues to disambiguate and improve distance perception, but that the degree to which they incorporate haptic size information is lower than the ideal observer prescribes. We also conclude that the distance responses are best explained as a process of drawing several samples from the posterior distribution over distance given sensations, and averaging them to form a distance estimate. This behavior is broadly consistent with a Bayesian perceptual inference model in which mistaken generative knowledge about haptic cues is used, and beliefs about distance are accessed by drawing samples from an internal posterior distribution.

The brain's use of sensory cues for disambiguating others has been reported in a variety of perceptual domains, and broadly falls under the category “perceptual constancy”. Constancy effects, like the present distance constancy, involve situations in which an observer cannot unambiguously estimate a scene property due to confounding influences from other “nuisance” properties, and so leverages “auxiliary” cues (in this study, haptic size) to rule out inconsistent possibilities. Auxiliary disambiguation effects, like constancy, have other names in the literature, like “cue promotion” [22], “simultaneous contrast” [23], and “taking-into-account” [24]. Many studies have reported “size constancy”, distance cues disambiguating object size perception [25]–[32], so it is not entirely surprising that size cues can conversely disambiguate distance perception.

Humans underestimating non-visual cue reliabilities and thus integrating them less strongly has been measured before by [10], [32]. There are several potential reasons for this phenomenon, one idea that has recently garnered support [33]–[36] is that sensory cues are used in accordance with their causal relationships to the unobserved scene properties: when the brain believes cues are unrelated to the desired scene property, it down-weights or outright ignores them. In the present study, this would mean the brain is unwilling to fully apply the haptic size cues because they might originate from a source independent of the ball, for instance imagine the hand touched a ball behind a photograph of a different ball; of course, such miscorrespondences are uncommon in nature, but examples like “prism adaptation” demonstrate the brain can accommodate and recalibrate in such situations. Another possibility is non-visual cues to spatial properties may be experienced far less frequently in life, and had fewer opportunities on which to be calibrated, so they are mistrusted.

Our finding that all our observers' responses are best modeled as sampling the posterior is consistent with recent studies and ideas about the representation and computation of probability in the brain. Using posterior sampling to generate responses in a choice task should manifest as probability matching of the options, a common finding in many behavioral tasks, including a perceptual audio-visual cue-combination task [13]. Sampling has also been used to provide a novel explanation for perceptual switching to multistable displays [11], [37]. Moreover, sampling provides an interpretation of neural activity in population codes and makes difficult probabilistic computations simple to neurally implement (see review by [38]).

Although Bayesian decisions are usually modeled as maximizing the posterior, maximization is not the best decision rule in all instances. MAP's optimality depends on both the task and the veridicality of the decision maker's posterior distribution. MAP assumes the decision maker's goal is to maximize the number of correct responses and that the posterior is based on the correct generative model for the data. When the posterior is not correct, basing responses on sampling provides exploration that can be used to improve the decision maker's policy. This idea has been extensively explored within reinforcement learning, where exploration is frequently implemented using a softmax decision strategy [39] where choices are stochastically sampled from an exponentiated distribution over the values of a set of discrete options. This idea can be generalized to the case of continuous decision variables. The value of an estimate is based on the reward function for the task. In our decision task, participants were “correct” whenever their choices fell within a narrow region relative to their posterior distribution. Approximating the experimental reward function as a delta function, the optimal strategy is to maximize the posterior. However, if we need to improve our estimate of the posterior, then it is important to estimate the error. Sampling from the posterior gives a set of values that can be used to compute any performance statistic, making it a reasonable strategy when an observer is needs information needed to learn - i.e. to assess and improve performance.

Though our models posit observers draw 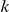 samples directly from the posterior and averaging, any decision rule that is sensitive to the posterior variance may produce similar predictions – for instance, it is possible that participants internally exponentiate the posterior and draw exactly one sample (detailed in Results). This means that for greater exponents, the posterior is more greatly sharpened; as the exponent approaches infinity, the posterior approaches a delta function located at the MAP estimate (after re-normalizing). This is a general strategy used in many machine learning domains to transition neatly between posteriors, MAP estimates, and “watered down” versions of the posterior. However we find this account unappealing because it implies that drawing more than one samples is less attractive to the observer's underlying perceptual mechanics than performing posterior exponentiation. Also, though our models assume posterior sample-averaging is a source for behavioral response variance (Figure 4), another possibility is that observers have uncertainty in the parameter values that characterize their generative knowledge itself, and actually draw samples of generative parameters instead of using deterministic parameter estimates. For instance, when combining haptic cues they may sample from an internal distribution over haptic reliability (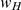). This could be a strategy for learning when the brain is uncertain about internal generative model parameters; because the observer receives feedback, and presumably wishes to calibrate the internal perceptual model, varying behavior by using different samples of internal model parameters avoids redundant feedback associated with similar behavioral responses to similar input stimuli.

Using a full probabilistic model of observers' sensation, perception, and decision-making processes provide us with answers to the four key questions we posed in the Model section. This study's analysis of data reported by [4] resulted in a much more comprehensive account of the computations responsible for distance and size perception. By formally characterizing a set of principled computational perception hypotheses, and choosing the best theoretical account of the measured phenomenology using Bayesian model selection tools, we demonstrated the power, robustness, and flexibility of this coherent framework for studying human cognition, and obtained deeper understanding of distance perception.
